# Preparation and Characterization of Cellulose Filled With Hydroxyapatite Biocomposite Film

**DOI:** 10.1002/bip.70038

**Published:** 2025-06-28

**Authors:** Ene Awodi, Turup Pandurangan Mohan, Kanny Krishnan

**Affiliations:** ^1^ Department of Mechanical Engineering, Faculty of Engineering and the Built Environment Durban University of Technology Durban South Africa

**Keywords:** biocomposite films, bioplastics, biopolymers, cellulose, fish‐scale hydroxyapatite

## Abstract

The packaging industry remains largely dominated by non‐degradable synthetic materials, raising environmental concerns and prompting increased interest in sustainable alternatives. As a result, biopolymers such as starch and cellulose have gained considerable attention. The present study investigates the thermal, mechanical, and hydrophilic properties of regenerated cellulose film as a potential eco‐friendly packaging material. The biopolymers utilized in this study were derived from secondary biowaste sources. The presence of transmittance bands corresponding to calcium and phosphate groups in the FTIR spectra, as well as the results of elemental composition analysis (EDX), confirmed the elemental makeup of the particles. FTIR analysis further revealed significant interactive bonding between the hydroxyl groups in the cellulose matrix and the calcium components of the FSHAp fillers. These interactions resulted in shifts in the IR transmittance bands in the biopolymer composite films. The incorporation of FSHAp fillers into the cellulose matrix enhanced the thermal stability of the cellulose films, with an observed improvement of 24%. At a filler concentration of 3 wt%, the char residue was 74.89% higher than that of the unfilled cellulose film. Additionally, the cellulose film containing 2 wt% FSHAp exhibited a tensile strength of 23 MPa, representing a 30% increase compared to the unfilled sample. This study introduces a novel biopolymer composite film as a promising sustainable and eco‐friendly alternative to conventional plastic‐based packaging materials. Furthermore, it supports the principles of the circular economy by offering a viable solution for managing abundantly available biomass waste.

## Graphical abstract

Processing sequence of biocomposite film.

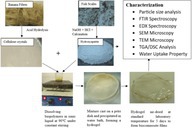

## 1 Introduction

Plastics have significantly enhanced modern life by contributing to the development of essential items such as automobiles, mobile phones, computers, and medical equipment, undoubtedly making daily life more convenient. However, this widespread utility has also led to the pervasive integration of plastics into nearly every aspect of human activity. Fossil‐derived plastics are particularly favored due to their desirable properties, including excellent barrier performance, rigidity, flexibility, and low production cost, which explains their extensive usage across various industries [1]. However, the improper management of plastic waste at the end of its life cycle has contributed to a growing global waste management crisis. According to Geyer et al. [2], only approximately 9% of non‐biodegradable plastic waste is recycled, 12% is incinerated, while the remaining 79% accumulates in landfills or is discarded into the natural environment. Furthermore, plastic waste has been identified as a major source of microplastics [3], which have been associated with various health conditions and reduced life expectancy. The accumulation of microplastics in the environment poses serious concerns for both human health and ecological systems [4]. Widespread reliance on plastic food containers, plastic‐coated metals and paper cartons, disposable cups, plastic bottles, and infant feeders contributes to the release of microplastic particles into food products and eventually into the body system. Plastics especially at the end of their life cycle, are subjected to environmental factors such as friction, temperature fluctuations, sunlight, pressure, and moisture, which cause them to break down into smaller fragments, ultimately leading to the formation of microplastics [5]. The first study to provide evidence of microplastics in the human placenta was published in 2021. In this study, six placentas were collected from pregnant women, and a total of 12 microplastic fragments, ranging in size from 5 to 10 μm, were detected across all samples [6]. Since then, multiple studies have reported the presence of microplastics in various human biological fluids, including blood [7], semen [8], and other body fluids [9], with findings suggesting potential disruptions to several physiological functions. Menezes et al. [10] observed that the frequency of micronucleated cells in freshwater fish increased with higher concentrations of microplastics, indicating the potential genotoxic effects of microplastics and their detrimental impact on fish health. Similarly, another study reported that the presence of microplastics in agroecosystems may exacerbate risks to plant development and, by extension, human health. Specifically, the combined presence of microplastics and arsenic was found to impair rice plant growth by inducing oxidative stress in the plants [11]. This suggests that plastic pollution poses not only a threat to human health but also has significant adverse effects on ecosystems, including aquatic and terrestrial wildlife. In light of the growing emphasis on sustainability, the development of eco‐friendly alternatives to conventional plastics should be prioritized.

Single‐use, fossil fuel‐based packaging materials currently dominate the global packaging industry, accounting for the largest share of packaging applications [12]. This trend has been partly attributed to the impact of the COVID‐19 pandemic, which influenced consumer preferences toward plastic packaging due to concerns over food safety [13, 14]. Common single‐use packaging films are typically produced from plastics such as polyethylene, polystyrene, polypropylene, and polyethylene terephthalate. Unfortunately, conventional packaging materials persist in the environment for extended periods, during which they leach harmful substances into the soil and nearby water bodies. Over time, environmental weathering further degrades these materials into microplastics [15]. This escalating crisis underscores the urgent need to transition from conventional packaging films to environmentally friendly alternatives, such as biopolymer‐based films. Recent studies have demonstrated the successful conversion of biopolymers including cellulose, gelatin, chitosan, and starch into promising packaging materials [16, 17]. The adoption of these biopolymers not only reduces reliance on petroleum‐based plastics but also facilitates the production of compostable and biodegradable packaging solutions. Biopolymers are derived from a diverse array of sources, including waste biomass as well as the cultivation of specific crops such as corn, potatoes, and straw.

Recent research trends have increasingly emphasized the use of waste biomass as a feedstock for the production of biopolymer composite films in packaging applications. This shift is motivated by both economic and environmental considerations. Traditional biopolymer sources, such as cassava and corn, raise ethical concerns due to their competition with arable land and food resources [18]. In contrast, waste biomass including food waste, agricultural residues, and organic industrial by‐products offers an abundant, cost‐effective, and sustainable alternative that aligns with the principles of the circular economy. A recent study investigated the aquaculture supply chain through the application of blockchain technology. The research addressed the challenge of valorizing fish waste by converting discarded materials into valuable resources. Additionally, the authors advocated for replacing single‐use conventional plastics with biodegradable packaging alternatives [19]. While this study specifically focused on the tuna fish industry, the associated waste management crisis extends to numerous other secondary sources suitable for biopolymer extraction. Fish scales are natural biopolymers primarily composed of hydroxyapatite and type I collagen. The utilization of fish‐derived biopolymers, such as collagen and gelatin, has been extensively documented in the literature. In contrast, fish scale hydroxyapatite has received limited attention regarding its potential applications beyond the biomedical field [20]. Due to their low commercial value, fish scales have limited practical applications despite being abundant biowastes that significantly impact solid waste management processes [21]. It is estimated that million tons of fish waste are discarded globally each year [20]. The upcycling of fish scales could play a crucial role in mitigating environmental pollution caused by this waste, while simultaneously generating added commercial value.

Tilapia is one of the most commonly farmed fish species in South Africa, where tilapia aquaculture is a rapidly expanding sector. This species is favored due to its high growth rate, tolerance to a wide range of temperatures and poor water quality, as well as its strong marketability and adaptability [22]. Tilapia fish scales are a valuable source of hydroxyapatite, a biologically important compound characterized by the presence of calcium and phosphate groups and known for its thermally stable phase. Compared to other calcium phosphates, hydroxyapatite derived from tilapia fish scales demonstrates superior thermal stability [23]. Previous studies have identified carbon, oxygen, calcium, and phosphorus as the primary elemental components of tilapia fish scales [24]. Similarly, banana plants are among the most widely cultivated crops globally. In South Africa, banana plantations are prominent in regions such as KwaZulu‐Natal (Durban) and the coastal areas, collectively covering approximately 11,000 ha. These fruit crops are also exported internationally [25]. Notably, the banana plant bears fruit only once, after which the entire plant is cut down. Approximately 88% of the plant's total weight consists of inedible parts, resulting in a significant portion of cellulose‐rich biomass remaining underutilized. Raw banana stem fibers contain around 48% cellulose, while delignified banana fibers exhibit a cellulose content as high as 79.1% [26]. Recent research has increasingly focused on converting waste biomass into polymer sources, including chitin from shrimp shells [27] and cellulose from bagasse [28]. However, some studies have relied on commercially sourced biomass [29] rather than engaging in direct extraction processes to develop biopolymer films for packaging applications.

Although the performance characteristics of biopolymer composite films such as tensile strength are often comparable to those of synthetic plastic counterparts, conventional polymers like low‐density polyethylene typically exhibit relatively low thermal stability [30]. The integration of eco‐friendly fillers has shown significant potential to enhance both the thermal stability and barrier properties of these films [31]. Thermal stability is a critical parameter for biopolymer films intended for food packaging and other applications, as it determines their capacity to endure processing conditions without melting or undergoing chemical degradation. Such degradation can compromise the mechanical, optical, and barrier performance of the films and may also lead to contamination of the packaged food. This consideration is particularly important because high temperatures are commonly applied during film manufacturing processes such as extrusion, molding, and casting. Numerous studies have explored the mechanical and barrier properties of biopolymer films. For example, cellulose has been blended with glycerol, citric acid, and gelatin, resulting in an enhancement of tensile strength from 4.5 to 7.73 MPa [32]. Similarly, the combination of cellulose derived from avocado seeds with gelatin crosslinked using citric acid has also been investigated [33]. In a recent study, the incorporation of lignin into regenerated cellulose films yielded a notably high tensile strength of 110.4 MPa, along with a water contact angle of 103.6° indicating improved hydrophobicity [34]. Furthermore, regenerated cellulose films have been reported to possess tensile strengths ranging from 32 to 77 MPa [26]. Despite these advancements, limited research has focused on the development of biopolymer films using fillers with inherently high thermal stability.

One of the primary challenges in processing biopolymers is their limited solubility in water and most conventional organic solvents. Room‐temperature ionic liquids (RTILs) have emerged as effective non‐derivatizing solvents capable of dissolving a variety of biopolymers, including cellulose. For instance, ionic liquids have been successfully utilized in the processing of gelatin/carboxymethyl cellulose films [35], starch‐based materials [36], and chitosan composites [37]. Although ionic liquids are relatively expensive, their high recyclability, often achieving recovery rates of up to 95%, enhances their sustainability [38]. Prior studies have demonstrated that rotary evaporation can be used to remove water from ionic liquids in the coagulation bath, with freeze‐drying employed to eliminate residual moisture [26, 39]. Furthermore, research by Jia et al. [40] confirmed that recycled ionic liquids can be reused multiple times, with only minimal differences observed between the original and recovered solvents.

The primary objective of this study is to develop a cost‐effective and environmentally friendly method for producing cellulose‐based films from low‐value agricultural waste, while incorporating bio‐fillers to enhance the thermal and mechanical properties of the resulting biocomposite films. Previous studies have shown that biopolymer films typically exhibit significant mass loss during thermal degradation. For instance, Ren et al. [36] reported an approximate 80% mass loss in starch‐IL films, and a similar observation was recorded by Singh et al. [41] for gelatin/carboxymethyl cellulose films. Additionally, a mass loss of around 70% was observed in polylactic acid/starch‐cellulose composites [42]. In this context, the present study explores the thermal stability of fish scale‐derived hydroxyapatite (FSHAp) and its potential application in the development of novel biopolymer composite films with enhanced thermal stability and tensile strength. Beyond demonstrating the functional potential of hydroxyapatite extracted from fish scales, this research contributes to waste valorization by addressing fish scale disposal challenges while offering economic value. In this work, cellulose was extracted from banana fibers via acid hydrolysis, and the resulting cellulose was dissolved in 1‐ethyl‐3‐methylimidazolium acetate to form cellulose films. FSHAp, extracted from tilapia fish scales, was incorporated as a bio‐filler. The study investigated the individual and interactive effects of the film components on thermal, mechanical, and hydrophilic properties using Fourier Transform Infrared Spectroscopy (FTIR), Thermogravimetric Analysis (TGA), Scanning Electron Microscopy (SEM), Energy Dispersive x‐ray Spectroscopy (EDX), tensile testing, and water contact angle measurements. The results of these analyses are presented and discussed in this article.

## 2 Experimental Procedure

### 2.1 Materials

Fresh tilapia fish scales were collected from a local fish market in Durban, South Africa. All chemicals used in this study were of analytical grade. Hydrochloric acid (HCl, 35% concentration) was obtained from Merck Group South Africa, and a 5 wt% sodium hydroxide (NaOH) solution was supplied by Shalom Laboratory Supplies. The ionic liquid 1‐ethyl‐3‐methylimidazolium acetate ([Emim][OAc], CAS 143314‐17‐4, ≥ 95.0% purity, HPLC grade) was purchased from Sigma‐Aldrich and used without further purification.

### 2.2 Methods

#### 2.2.1 Extraction of Fish‐Scale Hydroxyapatite

Tilapia fish scales were thoroughly washed with distilled water to remove blood, dirt, and residual skin membranes. This washing process was repeated until the scales were visibly clean. The cleaned scales were then air‐dried under standard laboratory conditions for 24 h. Hydroxyapatite was extracted from the dried tilapia fish scales following previously established methods [23, 43, 44]. Briefly, the dried scales underwent a deproteinization pre‐treatment by soaking in 1 N hydrochloric acid (35%) at 28°C for 24 h, using a liquor‐to‐scale ratio of 2:1. After filtration, the scales were rinsed three times with distilled water. This was followed by an alkaline treatment, in which the scales (1:2 w/v) were stirred in 1 N sodium hydroxide solution for 12 h at 28°C. The resulting white precipitate was filtered and washed repeatedly with distilled water until a neutral pH was achieved. The neutralized product was oven‐dried at 70°C for 3 h. The brittle, dried scales were then ground using a tabletop mortar to obtain non‐uniform powder particles. Subsequently, 30 g of the ground material was placed in a 250 mL bowl of a Retsch PM 100 planetary ball mill (hardened steel) and milled for 2 h at 550 rpm using 30 stainless steel balls (10 mm diameter) to achieve smaller particle size. A ball‐to‐powder ratio of 1:1 was maintained during milling. Thirty grams of the powdered sample was placed in alumina crucibles and subjected to calcination in a muffle furnace (MX813 TP). The calcination process was conducted at a heating rate of 5°C/min up to 800°C and maintained for 2 h. The sample was then allowed to cool gradually inside the furnace at a controlled cooling rate of 5°C/min to room temperature [45, 46].

#### 2.2.2 Preparation of Biopolymer Composite Films

The biopolymer composite films were prepared using a modified solvent casting technique, based on the method described by Pang et al. [47]. In this study, 9 wt% of 1‐ethyl‐3‐methylimidazolium acetate was placed in a beaker and heated to 90°C under continuous stirring at 3000 rpm. Fish scale hydroxyapatite (FSHAp) fillers were added to the ionic liquid in varying concentrations (1, 2, and 3 wt%) and stirred for 1 h to ensure uniform dispersion. Preliminary investigations revealed that a 2 wt% cellulose concentration demonstrated optimal film‐forming properties. It was also observed that the viscosity of the solution increased with cellulose content, potentially hindering filler dispersion and matrix–filler interaction. Therefore, to ensure uniformity and avoid processing issues such as poor dissolution and inadequate interfacial adhesion, the cellulose content was fixed at 2 wt% for all film formulations. Additionally, filler loading beyond 3 wt% resulted in a highly viscous paste that could not be properly cast on a Petri dish, thereby setting 3 wt% as the upper limit for filler incorporation. The resulting solution is cast onto a Petri dish and immediately immersed in a distilled water bath at 30°C for 1 h to facilitate film formation. The resulting hydrogels were subsequently air‐dried under ambient laboratory conditions for 5 days to yield the final biopolymer composite films.

## 3 Characterization

### 3.1 Particle Size Analysis

The average particle size of the fish scale powder was determined using an Anton Paar Particle Size Analyzer (PSA 1190). The powdered sample was introduced into the sample holder, which was mounted on a vibrational platform to ensure consistent dispersion. Compressed air and a Venturi nozzle were employed to disperse the particles uniformly. As the particles passed through a laser beam, the instrument measured the diffraction patterns produced. These diffraction patterns were then analyzed by the accompanying software, which processed the data in real time and displayed the particle size distribution on the connected computer interface.

### 3.2 Fourier Transform Infrared (FTIR)

To analyze the functional group composition of the particles and the prepared biopolymer composite films, Fourier Transform Infrared Spectroscopy (FT‐IR) was employed. Characterization was conducted using a PerkinElmer Spectrum 3 spectrometer. The spectra were recorded in the wavenumber range of 400–4000 cm^−1^, with a resolution of 2 cm^−1^. The resulting FT‐IR spectra were presented as plots of transmittance versus wavenumber, enabling the identification of characteristic functional groups present in the samples.

### 3.3 Energy Dispersive X‐Ray Spectroscopy (EDX)

Energy Dispersive x‐ray Spectroscopy (EDX), an elemental analysis technique, was employed to determine the elemental composition of the material extracted from tilapia fish scales. This technique is based on the emission of characteristic x‐rays generated when a sample is bombarded with an electron beam, allowing for the identification and quantification of elements present. EDX analysis was conducted using a ZEISS EVO EDX system, providing insights into the relative abundance of each element within the sample.

### 3.4 Scanning Electron Microscope (SEM)

The morphology of the fish scale hydroxyapatite (FSHAp) and the surface characteristics of the prepared biopolymer composite films were examined using a ZEISS EVO scanning electron microscope.

### 3.5 Thermal Analysis

Thermogravimetric analysis (TGA) was employed to investigate the thermal behavior of the prepared films. Changes in sample properties were recorded as a function of increasing temperature at a constant heating rate of 5°C/min using the SDT Q600 instrument. Additionally, heat flow through the samples (DSC analysis) was measured, with the sample's heat flow per unit weight plotted against temperature.

### 3.6 Mechanical Analysis

Tensile testing of the biopolymer composite films was conducted using a LRX Tensile Testing Machine (Lloyd, USA) following the ASTM D882‐10 standard. Film samples were prepared with dimensions of 60 mm × 9 mm and tested with a gauge length of 25 mm at a crosshead speed of 10 mm/min. Typical stress–strain curves were generated to determine the tensile strength and tensile modulus of the developed biopolymer composite films. Each test was performed in quintuplicate, and the average values were reported.

### 3.7 Water Uptake Properties

#### 3.7.1 Swelling Index (SI)

The swelling index of the films was determined following the method described by Susmitha et al. [48] with slight modifications. Film samples were cut into 2 cm × 2 cm pieces, dried at 100°C for 12 h, and initially weighed (*W*_0_). The dried samples were then immersed in 50 mL of distilled water at 25°C for 5 min. After swelling, the samples were gently blotted with filter paper to remove surface moisture and reweighed (*W*_1_). The amount of absorbed water was calculated using Equation (1):

(1) $$\mathrm{SI}\;\left(\%\right)=\;\frac{W_{1}-W_{0}}{W_{0}}\times 100$$

where, SI (%) represents the swelling index, and *W*_0_ and *W*_1_ denote the weights of the dried and swollen film samples, respectively.

#### 3.7.2 Water Contact Angle

The water contact angle (WCA) test is used to evaluate the hydrophilicity or hydrophobicity of a surface. In this study, the contact angle measurements were conducted using the Ossila contact angle goniometer (Model L2004A1). A syringe filled with distilled water was used to dispense a droplet onto the surface of the biopolymer composite film mounted on the sample holder. An integrated camera captured the droplet profile upon contact with the surface. The associated PC software provided an intuitive interface for analyzing the contact angle and determining surface wettability characteristics.

#### 3.7.3 Water Absorption Kinetic

Water absorption kinetics were evaluated following the method described by Oluwasina and Awonyemi [49], with slight modifications. Biocomposite film samples were cut into 2 cm × 2 cm pieces and oven‐dried at 100°C for 2 h to determine their initial dry weight (*W*_0_). Each sample was then immersed in 50 mL of distilled water at 25°C. The weight gain of the samples was recorded at 5‐min intervals until equilibrium was reached. The water absorption kinetics were calculated using Equation (2):

(2) $$\text{Water Absorption}=\frac{W_{w}-W_{d}}{W_{d}}\times 100$$

where, $W_{w}$ denotes the weight of wet film and $W_{d}$ the weight of dry film sample.

#### 3.7.4 Water Solubility (WS)

The water solubility of the film samples was evaluated following the method described by Moradi et al. [50], with slight modifications. Initially, the samples were dried in an oven at 100°C until a constant weight was achieved, and the initial dry weight was recorded. The dried films were then immersed in 50 mL of distilled water at 25°C for 24 h. After immersion, the films were gently blotted to remove surface moisture and subsequently re‐dried in an oven at 100°C to a constant weight. The water solubility was calculated using Equation (3):

(3) $$\text{Water Solubility}\;\left(\%\right)=\frac{{\mathrm{Dw}}_{i}-{\mathrm{Dw}}_{f}}{{\mathrm{Dw}}_{i}}\times 100$$

where, ${\mathrm{Dw}}_{i}$ denotes the initial dry weight of film sample prior to immersion in water and ${\mathrm{Dw}}_{f}$ represents the final dry weight of film sample after immersion, blotting and subsequent drying.

#### 3.7.5 Coefficient of Friction

The coefficient of friction test was conducted using a Labthink MXD‐02 testing apparatus at a crosshead speed of 10 mm/min and a sledge mass of 50 g. Each sample was tested five times, and the average values were recorded. Both the static and dynamic coefficients of friction were measured during the analysis.

## 4 Results and Discussion

### 4.1 Particle Size Analysis

**FIGURE 1:**
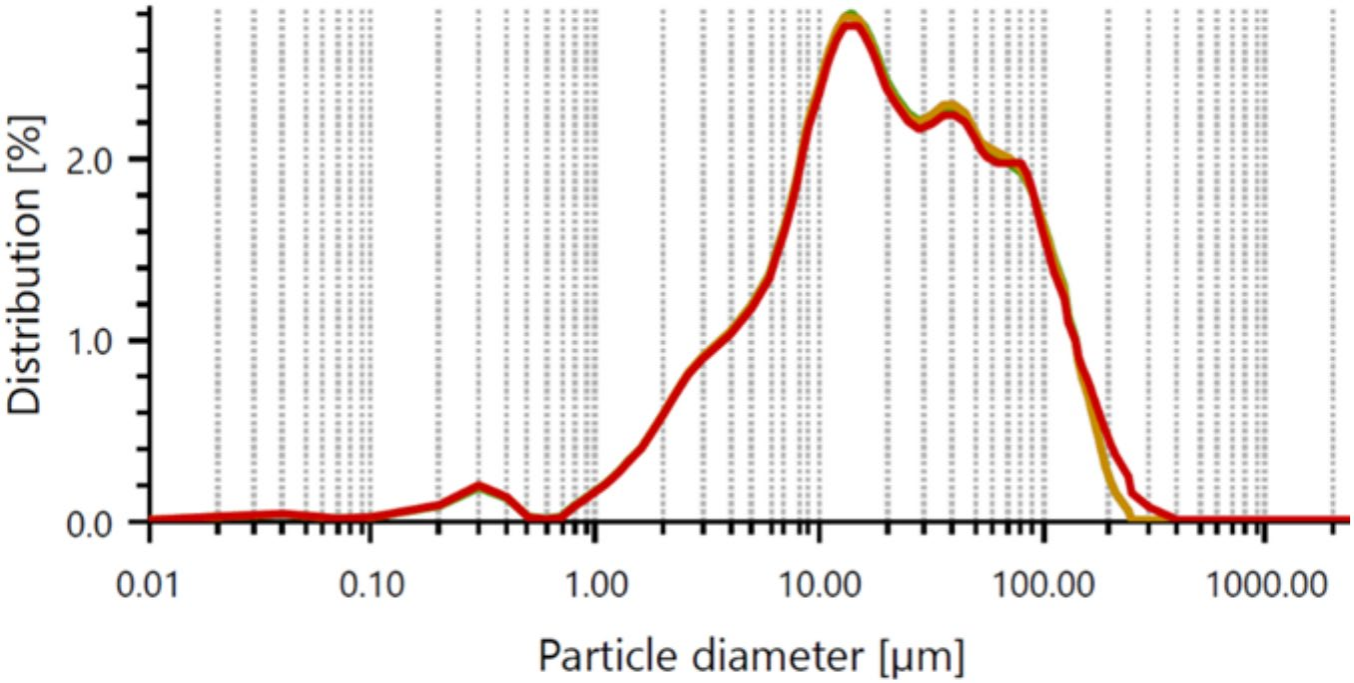
Particle size distribution of extracted fish scale hydroxyapatite.

**FIGURE 2:**
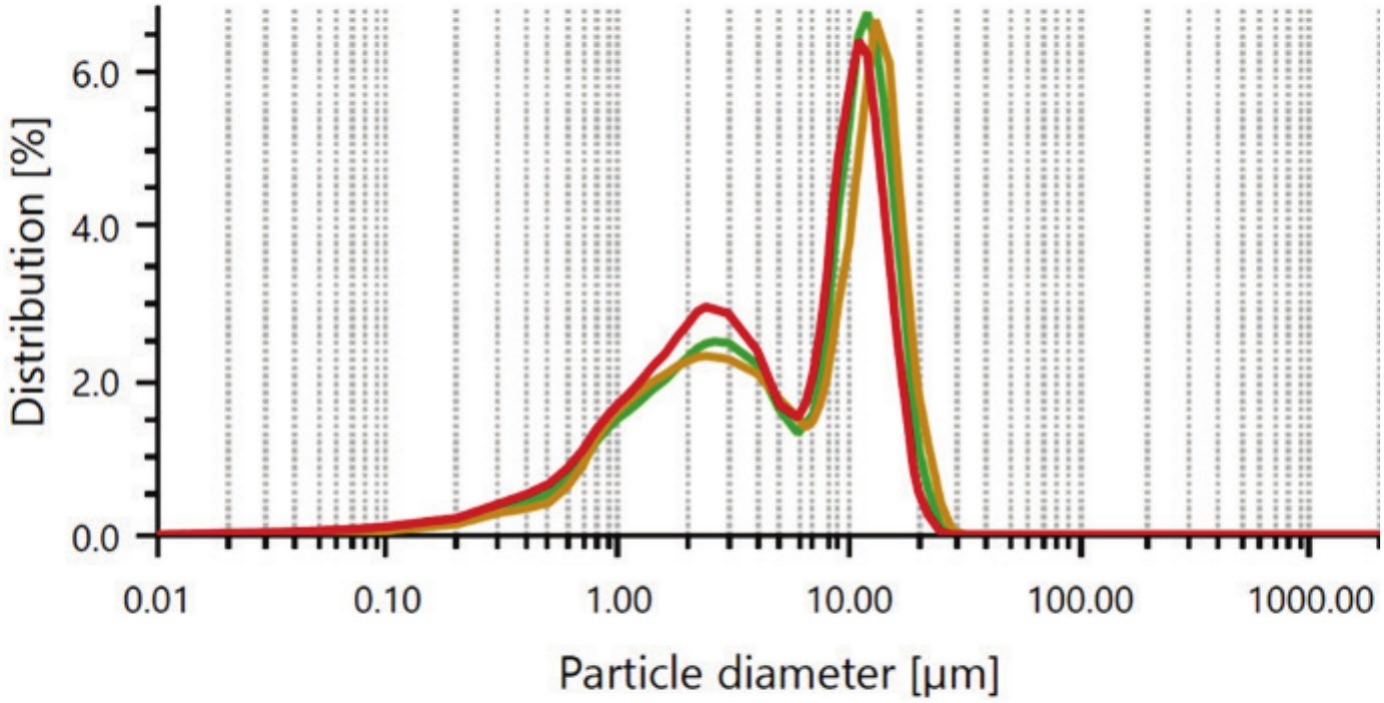
Particle size distribution of cellulose.

Figure 1 displays the particle size measurements and distribution percentages of the FSHAp particles, with the analysis software reporting a mean particle size range of 34–37 μm. Figure 2 illustrates the particle size distribution of cellulose extracted via acid hydrolysis, which was determined to be between 6.2 and 7.2 μm. Smaller reinforcement particles typically offer superior reinforcement capabilities due to their increased surface area, which facilitates more efficient stress transfer within the composite matrix [51]. Therefore, particle size is a critical factor influencing the performance properties of composite materials.

### 4.2 Fourier Transform Infrared (FTIR)

**FIGURE 3:**
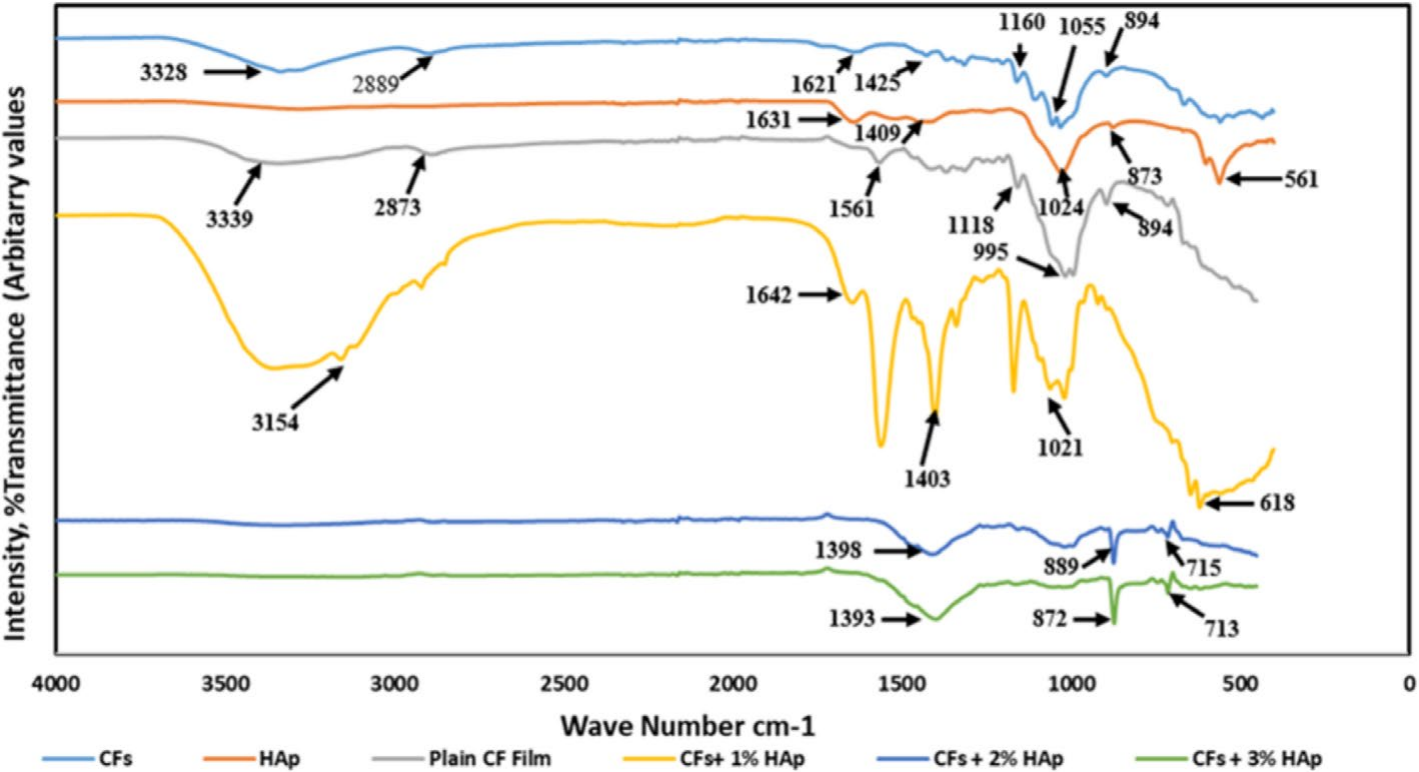
FTIR spectra of cellulose (CFs), hydroxyapatite HAp (FSHAp), unfilled regenerated cellulose film (plain CF film), and 1, 2, and 3 wt% FSHAp filled biopolymer composite films.

Figure 3 presents the FTIR spectra of extracted cellulose, FSHAp, and the developed biopolymer composite films, showing various absorption bands within the 400–4000 cm^−1^ range. Notably, the characteristic hydroxyl group peaks typically observed between 3200–3500 and around 630 cm^−1^ are absent. This absence may be attributed to the calcination process, which likely reduced the hydroxyl group content in the FSHAp. The v_3_ asymmetric stretching vibrations of phosphate groups are evident at 1024 cm^−1^ [52], while the peak at 561 cm^−1^ corresponds to the v_4_ bending mode of the phosphate group. Additionally, the transmittance peak observed at 873 cm^−1^ is ascribed to the v_2_ bending of carbonate groups, and a band associated with carbonate groups is evident at 1409 cm^−1^ [53, 54]. A peak at 1631 cm^−1^ corresponds to the v_2_ bending mode of adsorbed water molecules [55]. Overall, the FTIR analysis confirms that the particles extracted from tilapia fish scales in this study are consistent with hydroxyapatite.

The IR spectrum of the extracted cellulose displays a prominent peak at 3339 cm^−1^ corresponding to O—H stretching vibrations, while the band at 2889 cm^−1^ is attributed to symmetric and asymmetric C—H stretching from the aliphatic groups in the cellulose chain. A peak observed at 1621 cm^−1^ is associated with the bending mode of adsorbed water, while the CH bending transmittance band appears at 1425 cm^−1^. The C—O—C pyranose ring skeletal vibration characteristic of cellulose is evident at 1160 cm^−1^, with a shoulder at 1055 cm^−1^ representing C—O—C stretching. Additionally, the band at 894 cm^−1^ corresponds to the β‐glycosidic linkages between glucose units in cellulose. A noticeable shift of the hydroxyl vibration from 3328 to 3339 cm^−1^ was recorded in the extracted cellulose, attributed to hydrogen bond disruption. The IR spectrum of the regenerated cellulose film exhibited similar features to that of the extracted cellulose particles, indicating that 1‐ethyl‐3‐methylimidazolium acetate functions as a non‐derivatizing solvent in cellulose regeneration [38]. Given the abundance of hydroxyl groups in cellulose and the phosphate and calcium content in FSHAp, interactions are likely mediated via hydrogen bonding and electrostatic forces. Specifically, electrostatic interactions may occur between the negatively charged oxygen atoms in cellulose and the positively charged calcium ions in FSHAp. The IR spectra of the developed biopolymer composite films further support these interactions. A shift in the hydroxyl group band from 3328 cm^−1^ in the extracted cellulose spectra to 3154 cm^−1^ in the 1 wt% FSHAp‐filled cellulose film indicates hydrogen bonding between cellulose and FSHAp. The shoulder at 1055 cm^−1^ (C—O—C stretching) shifts to 1021 cm^−1^, suggesting partial hydrogen bond disruption. This also confirms that the macromolecular structure of cellulose remains intact in the biopolymer composite film [38]. A new band at 1642 cm^−1^ in the IR spectra of the 1 wt% FSHAp‐filled biopolymer composite film corresponds to the presence of available hydroxyl groups. The carbonate‐related transmittance band at 1409 cm^−1^ in pure FSHAp shows shifts to lower wavenumbers at 1403, 1398, and 1393 cm^−1^ for the 1, 2, and 3 wt% FSHAp‐filled films, respectively. Similarly, the carbonate v_2_ bending band at 873 cm^−1^ shifts to lower wavenumbers at 613, 715, and 713 cm^−1^ across the same filler concentrations. These shifts in transmittance intensity are consistent with findings from previous studies. For example, Azzaoui et al. [52] reported a similar trend in a study involving the preparation of a composite film from cellulose and hydroxyapatite, where interactive hydrogen bonds were formed between the hydroxyl groups of cellulose and the calcium ions of hydroxyapatite. Overall, the FTIR results confirm molecular interactions between the negatively charged hydroxyl groups in cellulose and the positively charged calcium ions in FSHAp, suggesting the formation of hydrogen bonds and electrostatic interactions within the biopolymer composite films.

### 4.3 Energy Dispersive X‐Ray (EDX)

**FIGURE 4:**
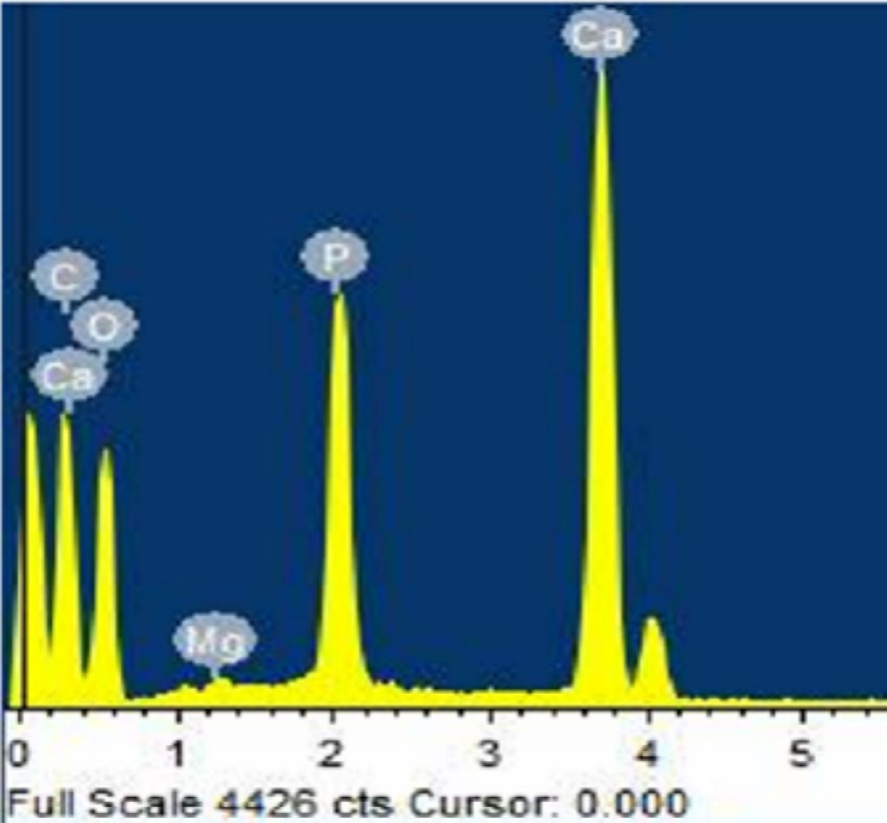
EDX of extracted FSHAp.

Figure 4a presents the Energy Dispersive x‐ray (EDX) analysis of the extracted FSHAp, indicating that the primary elements present are oxygen (40.91%), carbon (34.09%), calcium (17.17%), and phosphorus (7.57%). These elemental compositions are characteristic of hydroxyapatite and are consistent with findings from previous studies [56]. Notably, these values align closely with those reported by Nyambi et al. [24], who recorded oxygen at 27%, carbon at 19.8%, calcium at 27%, and phosphate at 11.9%, further validating the elemental composition of hydroxyapatite derived from biological sources. In addition, magnesium was detected as a trace element at a concentration of 0.29%. The presence of calcium and phosphorus in notable proportions confirms the successful extraction of hydroxyapatite from tilapia fish scales. This outcome supports the results obtained from the FTIR analysis, reinforcing the identification of the material as fish scale‐derived hydroxyapatite (FSHAp) (Table 1).

**TABLE 1:** Elemental composition of FSHAp.

Element	Weight %	Atomic %
Carbon (C)	34.09	46.69
Oxygen (O)	40.91	42.06
Magnesium (Mg)	0.29	0.20
Phosphorus (P)	7.5	4.02
Calcium (Ca)	17.5	7.04
Total	100	

### 4.4 Scanning Electron Microscope (SEM)

**FIGURE 5:**
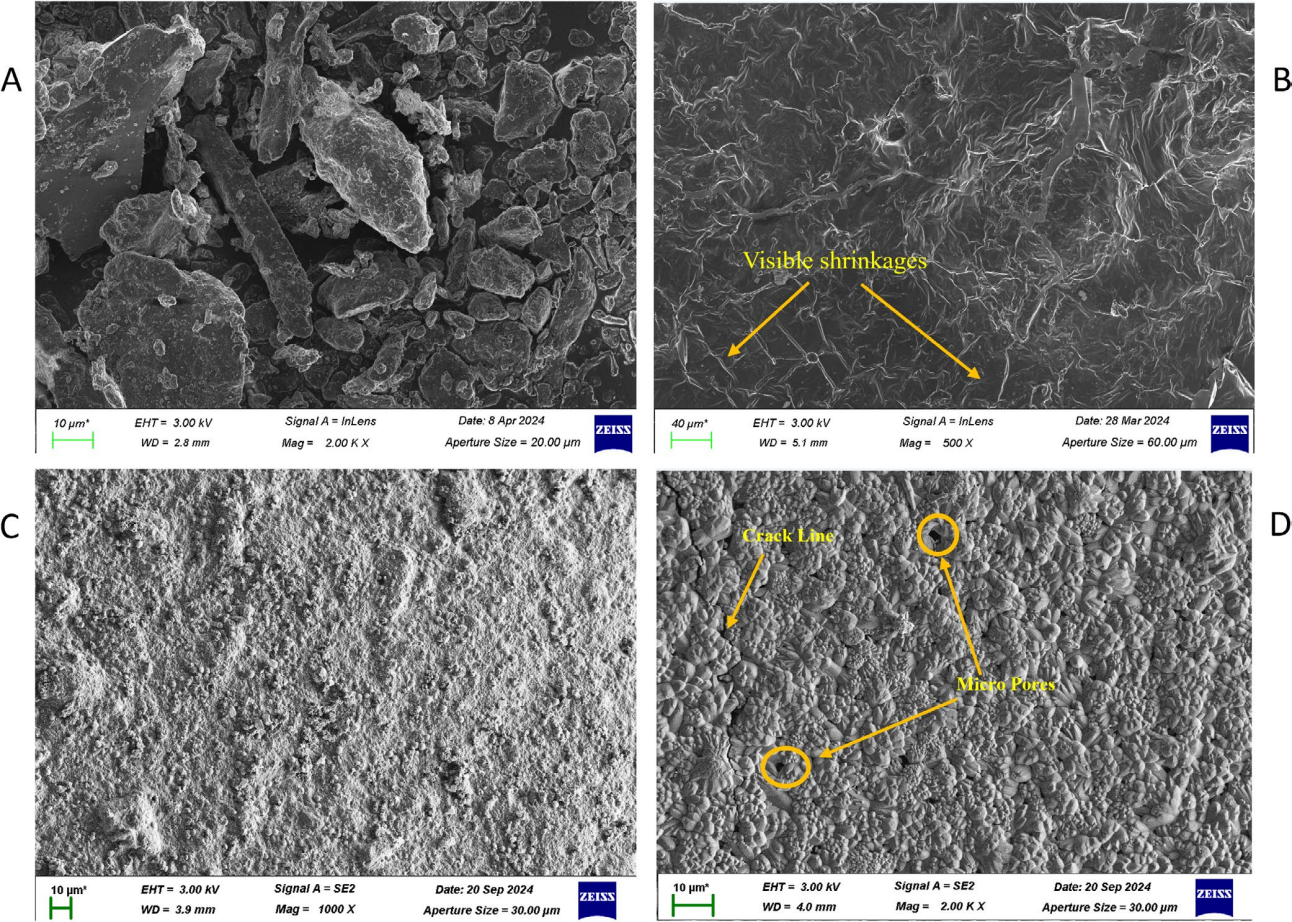
Scanning electron micrographs showing surface morphology of (a) fish scale‐derived hydroxyapatite (FSHAp), (b) biopolymer composite film filled with 1 wt% FSHAp, (c) biopolymer composite film filled with 2 wt% FSHAp, and (d) biopolymer composite film filled with 3 wt% FSHAp.

Figure 5a presents the scanning electron microscopy (SEM) image of fish scale‐derived hydroxyapatite (FSHAp), revealing an irregular polygonal morphology. The particles exhibited minimal agglomeration and a range of particle sizes, consistent with previous findings [56]. Additionally, the characteristic flat, plate‐like morphology typical of calcium phosphate compounds was observed, aligning with the report by Pon‐On et al. [57]. Figure 5b–d shows SEM micrographs of the biopolymer composite films filled with 1, 2, and 3 wt% FSHAp, respectively. As the concentration of FSHAp increased, the surface roughness of the films also increased, a trend similarly observed in earlier studies [52, 58]. The SEM image of the 1 wt% FSHAp‐filled film shows surface shrinkage, which may be attributed to the higher cellulose content (2 wt%) and lower FSHAp filler concentration at 1 wt%. Cellulose is known to exhibit shrinkage under drying conditions due to capillary stresses, as noted by Hasan et al. [17]. At 2 wt% FSHAp concentration, the film sample displayed increased surface heterogeneity and a denser microstructure, suggesting enhanced filler–matrix interaction. The 3 wt% FSHAp‐filled film (Figure 5d) exhibited the highest degree of heterogeneity, with visible microcracks and pores. This may be due to excessive filler concentration, which resulted in poor dispersion within the cellulose matrix. During processing, an increase in solution viscosity was observed, accompanied by a noticeable decrease in magnetic stirring efficiency. This likely led to insufficient incorporation of FSHAp into the cellulose matrix at higher loading levels. This outcome suggests that the ionic liquid used in this study, although suitable for dissolving cellulose even at concentrations up to 5 wt% (Pang et al. [47]), does not adequately dissolve FSHAp at 3 wt% concentration. Similar findings have been reported by Chowdhury et al. [59], who observed coarse granules on biopolymer film surfaces at higher filler concentrations (20 g berry starch in 60 g tamarind starch), and by Muhammad et al. [60], who investigated hydroxyapatite extraction from fish scales using ionic liquids. Additionally, fish scale particles are known to introduce a certain level of surface coarseness in biopolymer films [61], further complicating homogeneous dispersion especially as the concentration increases. Factors such as stirring time, temperature, and dissolution duration significantly influence filler miscibility. In this study, even after 1 h of stirring at 90°C, undissolved FSHAp remained in the solution, with the amount of undissolved material increasing in proportion to the FSHAp concentration introduced.

### 4.5 Thermal Analysis

**FIGURE 6:**
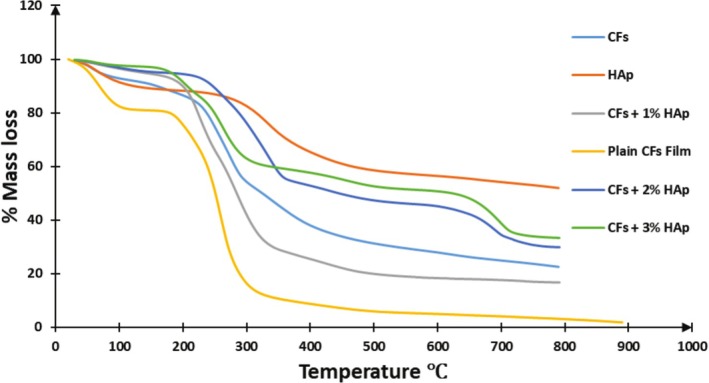
The thermogravimetric analysis curves of (a) cellulose (CFs), (b) fish scale‐derived hydroxyapatite (FSHAp), (c) regenerated cellulose film (plain CFs film), (d) biopolymer composite film filled with 1 wt% FSHAp (CFs+1% HAp), (e) biopolymer composite film filled with 2 wt% FSHAp (CFs+2% HAp), and (f) biopolymer composite film filled with 3 wt% FSHAp (CFs+3% HAp).

**FIGURE 7:**
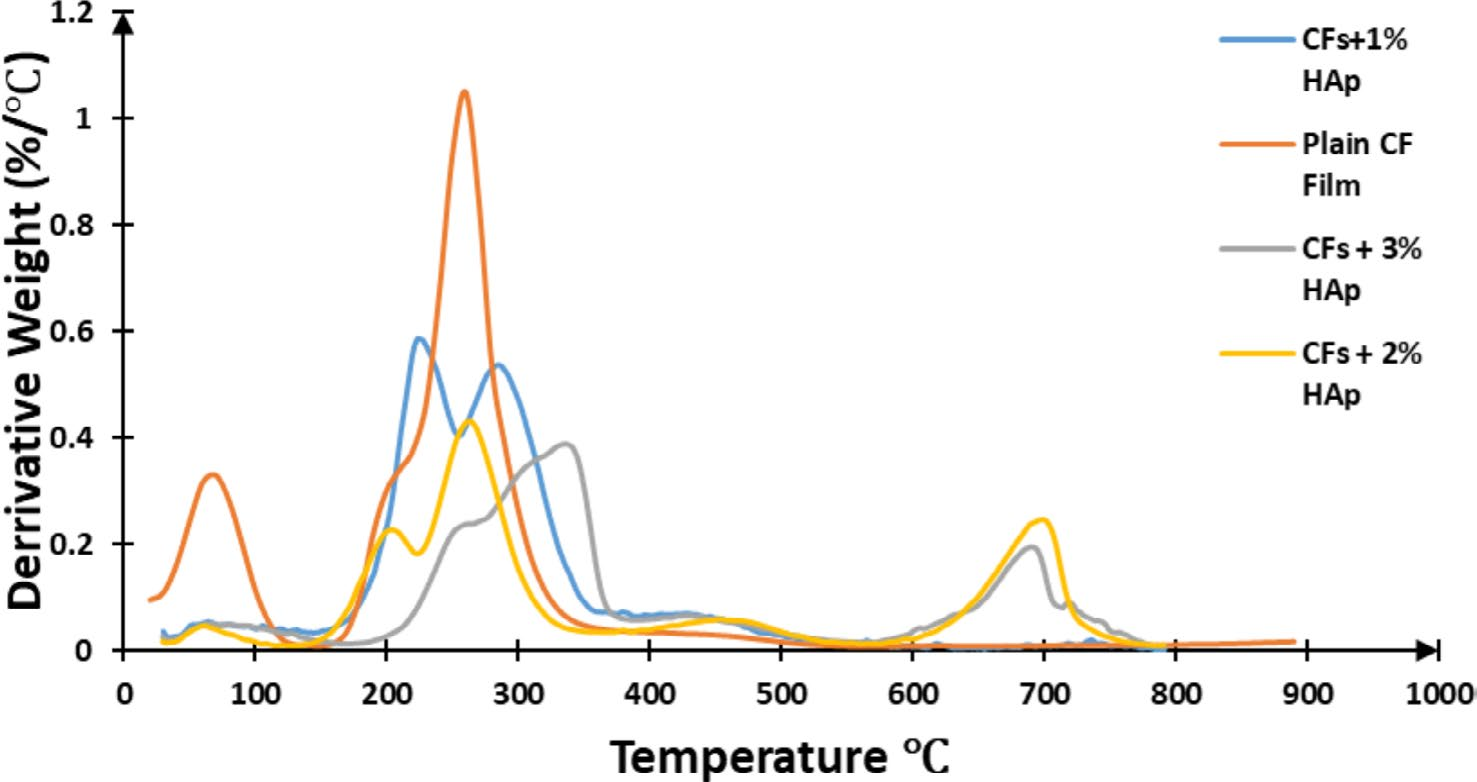
Differential thermogravimetry curves of (a) regenerated cellulose film (plain CFs film), (b) biopolymer composite film filled with 1 wt% FSHAp (CFs+1% HAp), (c) biopolymer composite film filled with 2 wt% FSHAp (CFs+2% HAp), and (d) biopolymer composite film filled with 3 wt% FSHAp (CFs+3% HAp).

**FIGURE 8:**
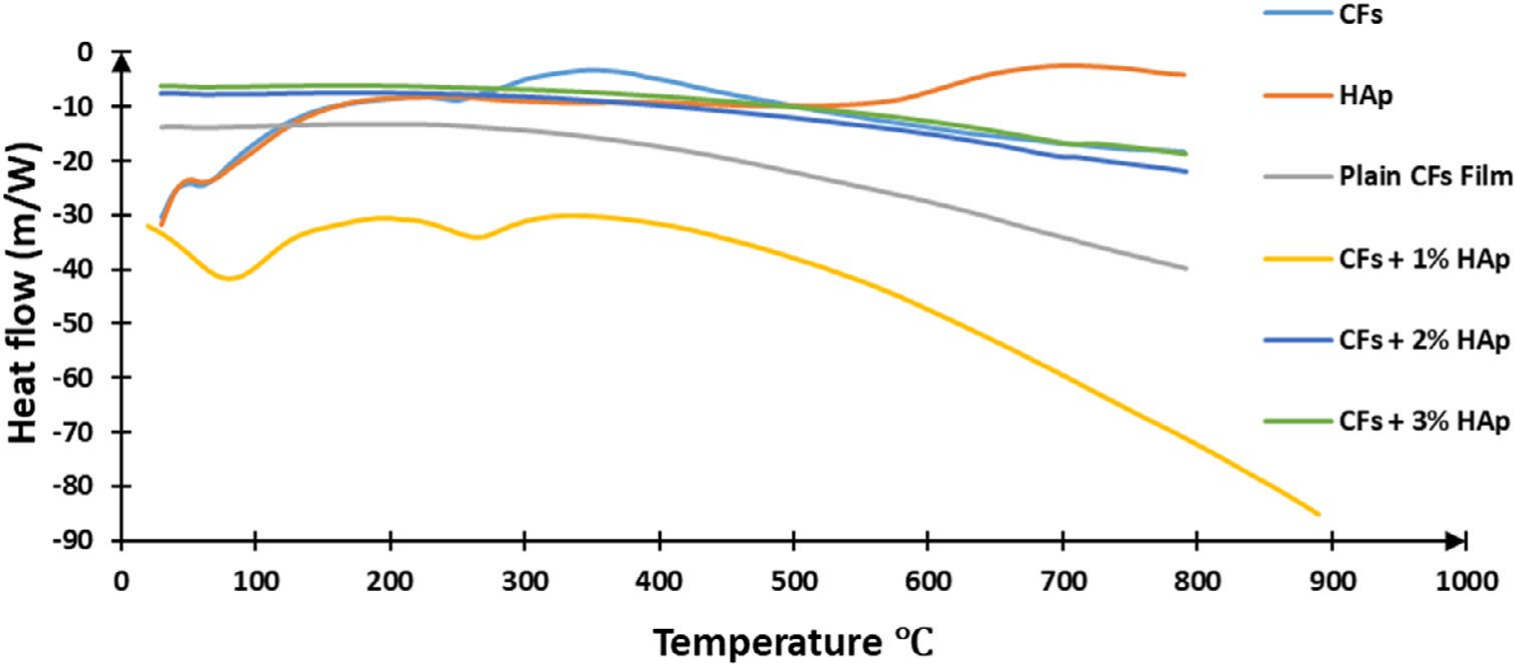
DSC curves of (a) regenerated cellulose film (plain CFs film), (b) biopolymer composite film filled with 1 wt% FSHAp (CFs+1% HAp), (c) biopolymer composite film filled with 2 wt% FSHAp (CFs+2% HAp), and (d) biopolymer composite film filled with 3 wt% FSHAp (CFs+3% HAp).

Thermogravimetric analysis (TGA) and derivative thermogravimetry (DTG), and Differential scanning calorimetry (DSC) were employed to assess the thermal stability and decomposition behavior of the biopolymer composite films by monitoring weight changes under a controlled temperature regime. The findings are presented in Table 2, Figures 6 and 7. Thermal stability in polymers reflects their capacity to withstand heat while retaining essential performance characteristics such as stiffness, strength, and elasticity. The thermal degradation profiles of hydroxyapatite extracted from fish scales (HAp), cellulose film (Plain CFs film), and the developed biopolymer composite films are depicted in Figure 7. The TGA of the regenerated cellulose film (plain CFs film) indicated a predominantly two‐step thermal degradation process. The initial weight loss occurred between 20°C and 100°C, primarily attributed to moisture evaporation. The major degradation phase occurred between 190°C and 310°C, resulting in a total mass loss of 85.78%. It was observed that the onset degradation temperature of the regenerated cellulose film began at approximately 190°C, which is lower than the onset degradation temperature of the extracted cellulose which was recorded at 238°C. This earlier onset is attributed to the amorphous structure developed during the regeneration process. This observation aligns with findings in previous studies [62, 63], which reported that regenerated cellulose typically exhibits a lower thermal degradation onset compared to its unregenerated counterpart. Fish scale‐derived hydroxyapatite (FSHAp) exhibited a single major degradation event between 310°C and 400°C, with a significant residual mass of 69.37% remaining at the end of the analysis (Figure 8).

**TABLE 2:** Provides a comprehensive summary of key thermal degradation parameters obtained from the thermogravimetric (TGA) and derivative thermogravimetric (DTG) analyses of the biopolymer composite films.

Film sample	TGA				DTG (°C)		
	$T_{\text{onset}}$ (°C)	$T_{\max}$ (°C)	Weight loss (%)	Char residue at 790°C (%)	$T_{\max1}$	$T_{\max2}$	$T_{\max3}$
Cellulose	100–220	238–310	77.43	22.57	—	—	—
HAp	—	310–400	30.63	69.37	—	—	—
Plain CFs	20–100	190–310	91	3.138	70	210	260
CFs + 1% HAp	100–215	219–344	70.32	16.71	—	212	280
CFs + 2% HAp	242–378	678–720	45.91	29.86	221	269	706
CFs + 3% HAp	198–302	681–734	65.55	33.33	265	342	710

*Note:* The table outlines the temperature range for the onset of decomposition, $T_{\text{onset}}$ and the temperature corresponding to the maximum rate of weight loss, $T_{\max}$ during thermal degradation. Additionally, the table presents the percentage weight loss occurring within the primary degradation window (300°C–400°C), the char residue remaining at 790°C, and the specific peak temperatures observed on the DTG curves $T_{\max1}$, $T_{\max2}$, and $T_{\max3}$.

Upon analysis of the biopolymer composite films, it was observed that the temperature at which the highest mass loss occurred (*T*_*max*_) corresponding to the primary thermal degradation of the biopolymers increased progressively with the increasing percentage of FSHAp filler incorporated into the matrix. For example, in the control film without any filler, significant decomposition, indicated by a sharp decline on the TGA curve, commenced at approximately 190°C. However, for the sample containing 1 wt% FSHAp, the onset of major thermal degradation shifted to 219°C. This trend continued with higher filler loadings: the 2 and 3 wt% FSHAp‐filled samples exhibited primary decomposition onset temperatures at 269°C and 342°C, respectively. This linear increase in thermal stability with filler content is an expected outcome, attributed to the presence of thermally stable FSHAp particles within the polymer matrix, which act as thermal insulators and retard the degradation process. Similarly, the residual mass recorded for each biopolymer composite film following the TGA analysis offers critical insights into char formation and the organic content of the films. A clear trend was observed: films with higher FSHAp filler content retained more residue post‐degradation. Specifically, char residues of 14.22%, 29.68%, 54.09%, and 62.64% were recorded for the regenerated (plain) cellulose film and the biopolymer composite films filled with 1, 2, and 3 wt% FSHAp, respectively. This progressive increase in char yield indicates enhanced thermal stability with increasing filler concentration, as the thermally stable FSHAp impedes the decomposition of the cellulose matrix and promotes char formation. Notably, the char residues obtained in this study are significantly higher than those reported in comparable studies. For example, Koçak Mutlu et al. [30] reported that zinc oxide‐doped low‐density polyethylene commonly used in packaging exhibited a char residue of less than 10% at 500°C. Similarly, in the work by Kodali et al. [64], force‐spun polycaprolactone with 5 wt% fish scale hydroxyapatite yielded only 4% char residue. Additionally, Azzaoui et al. [52] observed a char yield below 20% for their cellulose/hydroxyapatite composite. These comparisons underscore the superior thermal resistance and residue‐retention performance of the FSHAp‐filled biopolymer composites developed in this study.

The DTG curves provide additional insight into the decomposition behavior of the biopolymer composite films, offering more detailed information on the stages of thermal degradation. For the plain cellulose film, distinct decomposition peaks were observed at approximately 70°C, 210°C, and 260°C. The maximum decomposition temperature peak at 260°C corresponds to the degradation of the amorphous phase of cellulose [63]. Conversely, all FSHAp‐filled cellulose film samples exhibited well‐defined, distinguishable peaks within the range of 200°C–350°C. For the 1 and 2 wt% FSHAp‐filled films, lower temperature peaks at 212°C and 221°C, respectively, are attributed to the degradation of amorphous cellulose particles. Higher temperature peaks at 260°C and 280°C for the 1 and 2 wt% samples, respectively, are associated with the decomposition of the biopolymer matrix formed through hydrogen bonding between the amorphous cellulose and FSHAp fillers, as supported by FTIR analysis. Notably, the highest decomposition temperature peaks were recorded at 706°C and 710°C for the 2 and 3 wt% FSHAp‐filled films, respectively. These peaks correspond to the thermal degradation of the FSHAp fillers within the composite matrix. Overall, the thermal stability of the biocomposite film samples was directly influenced by the concentration of FSHAp filler, with higher filler loadings resulting in enhanced thermal resistance and elevated decomposition temperatures.

On the DSC curve of the cellulose, the heat flow demonstrated a transition from a lower energy state to a higher energy state, characterized by the release of energy, indicating an exothermic process occurring between 230°C and 300°C. This peak is associated with a change in endothermic behavior, indicating the transition from a glassy state to an elastic state [65]. The thermal stability of cellulose is influenced by the temperature at which hydrolysis is conducted; higher hydrolysis temperatures tend to dissolve more crystalline regions of cellulose, thereby reducing the thermal stability [66]. For the FSHAp particles, the melting point was observed around 600°C, significantly higher than the 250°C melting point of the cellulose. A shoulder around 100°C on the DSC curves of cellulose corresponds to the elimination of residual moisture content. The characteristic cellulose‐related depression between 200°C and 300°C, representing the melting temperature of cellulose, was also observed on the DSC curve of the biopolymer composite film sample at 1 wt% FSHAp filler concentration. However, this peak was absent in the samples with 2 and 3 wt% FSHAp filler loadings, suggesting enhanced structural changes, resulting in improved thermal interactions at higher filler concentrations. The heat flow versus temperature plots revealed that the regenerated cellulose film exhibited the lowest resistance to heat flow, in contrast to the biopolymer composite films with varying FSHAp filler concentrations. The 3 wt% FSHAp‐filled film demonstrated the highest resistance to heat flow, indicating improved thermal stability. In conclusion, both cellulose and FSHAp and the biopolymer composites exhibited a consistent trend in their thermal response, which is in alignment with the information obtained from the thermogravimetric thermograms and the char residues recorded.

### 4.6 Mechanical Properties

**FIGURE 9:**
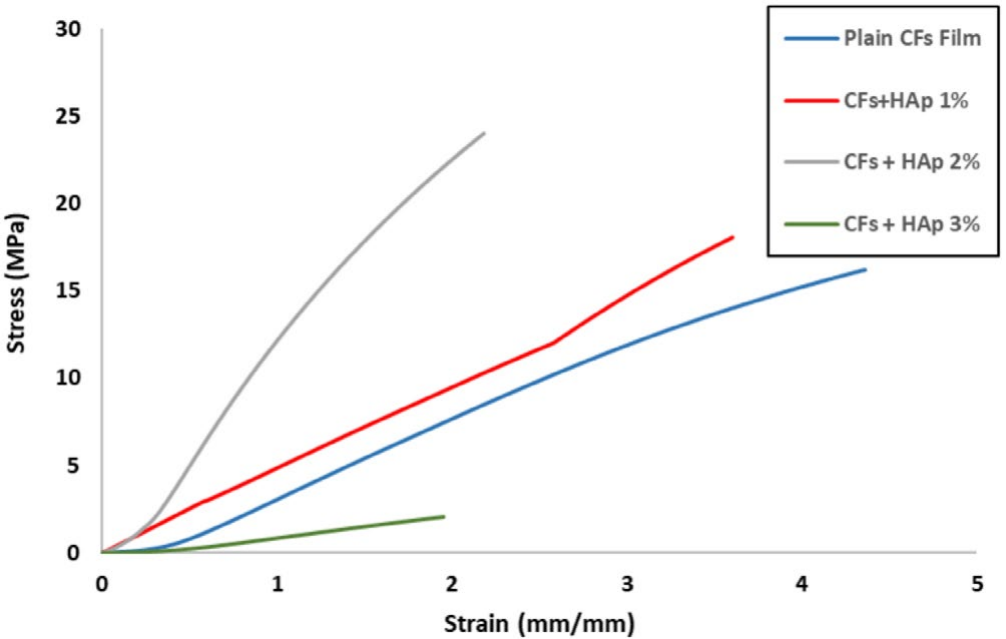
Stress–strain curve of (a) regenerated cellulose film (plain CFs film), (b) biopolymer composite film filled with 1 wt% FSHAp (CFs+HAp 1%), (c) biopolymer composite film filled with 2 wt% FSHAp (CFs+HAp 2%), and (d) biopolymer composite film filled with 3 wt% FSHAp (CFs+HAp 3%).

**FIGURE 10:**
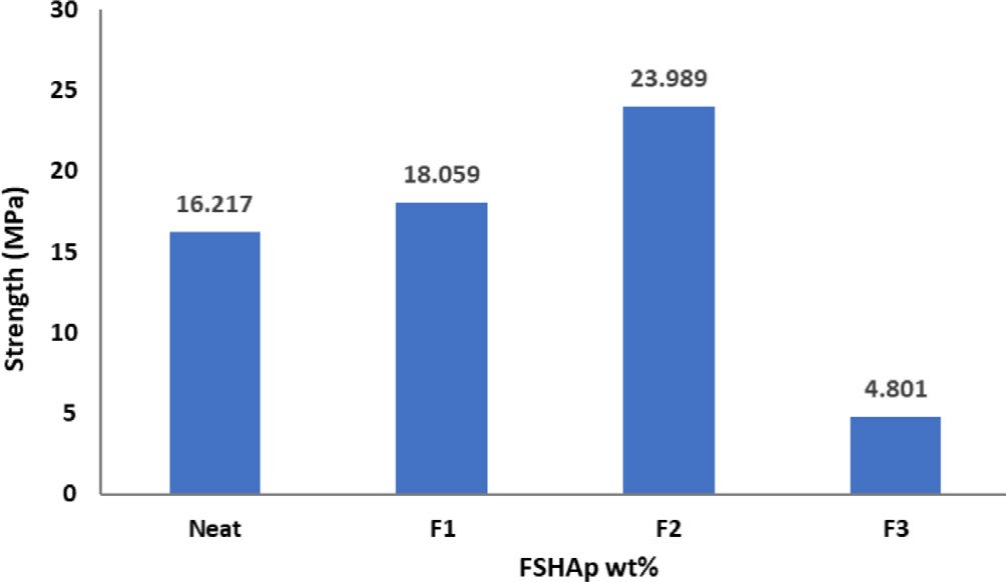
Tensile strength of (a) regenerated cellulose film (plain CFs film), (b) biopolymer composite film filled with 1 wt% FSHAp (F1), (c) biopolymer composite film filled with 2 wt% FSHAp (F2), and (d) biopolymer composite film filled with 3 wt% FSHAp (F3).

**FIGURE 11:**
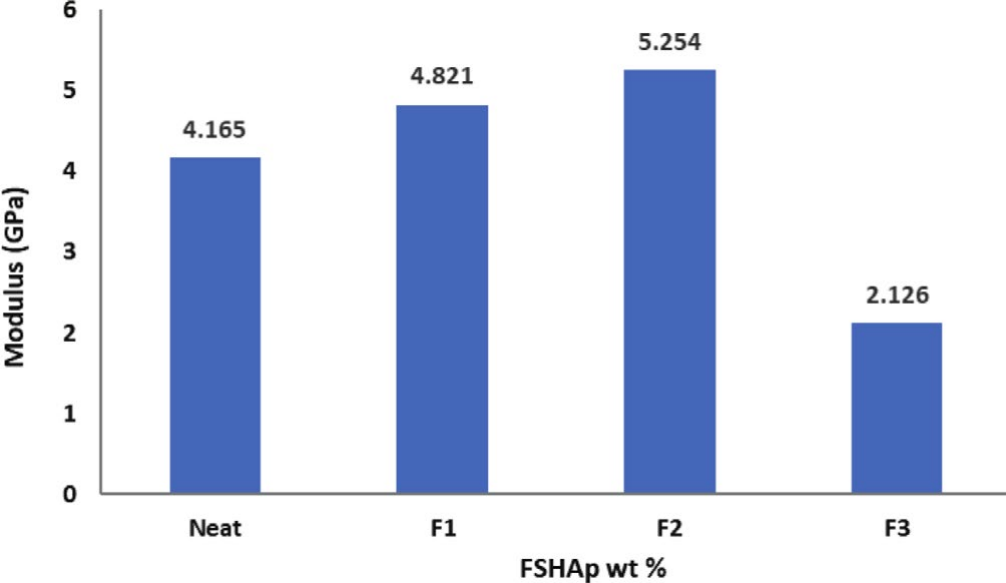
Tensile modulus of (a) regenerated cellulose film (plain CFs film), (b) biopolymer composite film filled with 1 wt% FSHAp (F1), (c) biopolymer composite film filled with 2 wt% FSHAp (F2), and (d) biopolymer composite film filled with 3 wt% FSHAp (F3).

The mechanical properties of biopolymer composite films are significantly influenced by their microstructure, which provides valuable insights into the internal architecture of these materials. The regenerated cellulose film (plain CFs film) exhibited a lower tensile strength compared to the 1 and 2 wt% FSHAp‐filled biopolymer composite films but showed a higher tensile strain, indicating superior ductility. In contrast, the addition of FSHAp fillers enhanced the stiffness of the films. Both matrix and filler concentrations play crucial roles in determining the mechanical behavior of the biopolymer composite films. Figure 9 illustrates the stress–strain curves for all developed biopolymer composite film samples. The incorporation of FSHAp contributed to notable enhancements in mechanical performance. The increase in tensile strength observed at 1 and 2 wt% filler concentrations suggests effective stress transfer within the film structure, likely due to improved interfacial bonding between the cellulose matrix and FSHAp fillers. This interaction is facilitated by hydrogen bonding between the calcium groups in FSHAp and hydroxyl groups in cellulose. Among the tested compositions, the biopolymer composite film with balanced proportions of 2 wt% cellulose and 2 wt% FSHAp achieved the highest tensile strength (Figure 10), indicating that optimal matrix–filler ratios enhance the mechanical strength of the composite. However, the film with 3 wt% FSHAp displayed a decline in tensile strength. This reduction corresponds with the morphological observations in the SEM micrograph (Figure 5d), where the film exhibited high heterogeneity and filler agglomeration, leading to phase separation and compromised mechanical performance. The decreased strength at higher filler loading is attributed to the reduced filler dispersion and inadequate matrix–filler interaction. Young's modulus results (Figure 11) showed a general trend of increasing stiffness with higher filler concentrations. However, the 3 wt% FSHAp sample recorded a drop in stiffness, with a tensile modulus of 2.12 GPa. The increased rigidity at this concentration contributed to the formation of surface crack lines (as seen in Figure 5d), thereby weakening the film due to poor stress distribution and transfer, hence the reduction in resistance to stress and ability to stretch. In contrast, the neat cellulose film and the 1 wt% FSHAp‐filled film exhibited tensile moduli of 4.165 and 4.821 GPa, respectively, while the 2 wt% FSHAp‐filled film achieved the highest tensile modulus of 5.254 GPa, an increase of 20.7% over the neat cellulose film. This confirms the reinforcing effect of FSHAp fillers at appropriate concentrations. When compared to similar materials in existing literature, the 2 wt% FSHAp‐filled film displayed tensile strength values comparable to polylactic acid (PLA), a widely used biopolymer for packaging, which has a tensile strength of approximately 25 MPa [31]. In contrast, other biopolymers such as starch typically exhibit tensile strengths ranging from 2 to 5 MPa [36, 67, 68]. In the current study, the tensile strength increased from 18 to 23 MPa with increasing FSHAp filler concentration of up to 2 wt%. Previous studies have also reported the influence of biopolymer concentration on film strength. For instance, the tensile strength of chitosan‐based CS/PVA/PEG films increased from 0.21 to 0.24 MPa when the chitosan concentration increased from 0.5 to 2 g [69]. Thus, the mechanical performance of the biopolymer composite films in this study demonstrates promising potential for replacing conventional fossil fuel‐based films in certain packaging and wrapping applications. Additionally, these regenerated films offer a sustainable alternative due to their biodegradability, being derived entirely from natural biopolymers.

### 4.7 Water Uptake Properties

#### 4.7.1 Swelling Properties of Biopolymer Composite Films

The solubility behavior of the biocomposite films is largely governed by the ability of water molecules to penetrate the polymer matrix and induce chain swelling [70]. In this study, the regenerated cellulose film (plain CFs film) was observed to be insoluble in water, consistent with previous findings [71]. However, upon immersion in water, biocomposite films underwent swelling due to water permeation into the cellulose matrix. The swelling index values presented in Table 3 indicate that the biopolymer composite films containing 1 and 2 wt% FSHAp fillers exhibited significantly lower swelling indices compared to the regenerated cellulose film and the film sample filled with 3 wt% FSHAp filler. This reduced swelling behavior at 1 and 2 wt% FSHAp filler concentrations is attributed to the porous nature of the FSHAp granules, which retained water within their interstitial spaces. In addition to this, the close interaction between the matrix and the fillers at 1 and 2 wt% FSHAp concentrations did not create free spaces in‐between for the free movement of water molecules through the structure of the films. This outcome aligns with the findings by Shalma et al. [61], who reported that a relatively uniform particulate nature of a biopolymer composite film impedes the accumulation of surplus water in the structure of the film. Whereas, the film with 3 wt% FSHAp filler displayed a markedly higher swelling index. This increase is associated with the formation of microcracks and pores in the film structure as a result of filler overload, which facilitated more rapid water ingress through the structure of the film sample as documented in Table 3. Overall, while the incorporation of FSHAp initially enhances the water resistance of cellulose‐based biocomposite, excessive filler loading compromises structural integrity, leading to increased swelling behavior due to microstructural disruptions.

**TABLE 3:** Swelling properties of biopolymer composite films.

Film sample	Initial dry weight (g)	Weight after 3 min	Weight after 5 min	Weight after 10 min
Plain CFs	0.150	0.270	0.440	0.530
CFs + 1% HAp	0.250	0.200	0.590	0.620
CFs + 2% HAp	0.330	0.560	0.620	0.697
CFs + 3% HAp	0.490	0.660	0.740	0.850

#### 4.7.2 Water Contact Angle

**FIGURE 12:**
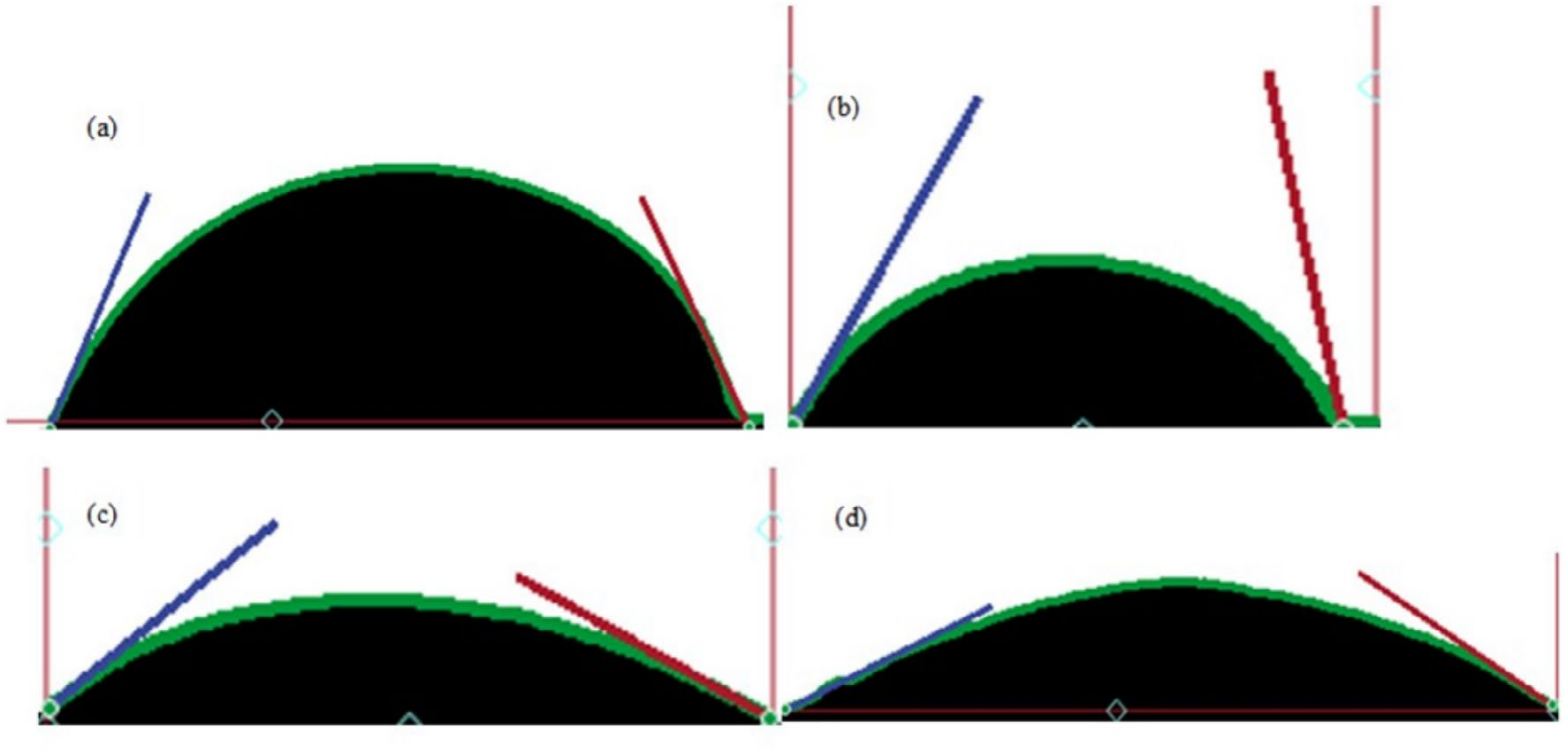
Water contact angle of (a) biopolymer composite film filled with 3 wt% FSHAp, (b) biopolymer composite film filled with 2 wt%, (c) biopolymer composite film filled with 1 wt% FSHAp, and (d) regenerated cellulose film (plain CFs film).

Figure 12a–d presents the water contact angle (WCA) images of the fabricated biopolymer composite films, while the corresponding quantitative data are summarized in Table 2. The water contact angle was measured using the sessile drop technique, which provides insight into the surface wettability of the materials. A contact angle below 90° typically indicates a hydrophilic surface, whereas higher values suggest a more hydrophobic or water‐repellent behavior (Xu et al. [72]). The results demonstrate a gradual increase in the contact angle with rising FSHAp filler content, indicating a decrease in surface wettability. The regenerated cellulose film (Plain CFs film) exhibited the lowest contact angle, confirming its inherently hydrophilic nature, consistent with prior findings on cellulose‐based films (Sridhar et al. [73]). Although all contact angles recorded remain below 90°, suggesting the films remain hydrophilic, the incremental increase reflects a reduced affinity for water on the film surfaces due to the FSHAp incorporation. This trend aligns with earlier reports in the literature. For instance, Rbihi et al. [74] reported a WCA of 17.4° for TiO_2_‐filled cellulose films, and Heredia‐Guerrero et al. [27] recorded a contact angle of 75° for ionic liquid‐regenerated chitin biopolymer. In comparison, the FSHAp‐filled films in this study demonstrated moderate hydrophilicity, and their water‐holding capacity improved as filler concentration increased. These findings are in agreement with observations by Kraemer et al. [75], further supporting the role of FSHAp in modifying surface energy and reducing film wettability on the surface (Table 4).

**TABLE 4:** Water contact angle of biopolymer composite films.

Film sample	Water contact angle (°)
Plain CFs	68
CFs + 1% HAp	73
CFs + 2% HAp	81
CFs + 3% HAp	87

#### 4.7.3 Water Absorption Kinetics

**FIGURE 13:**
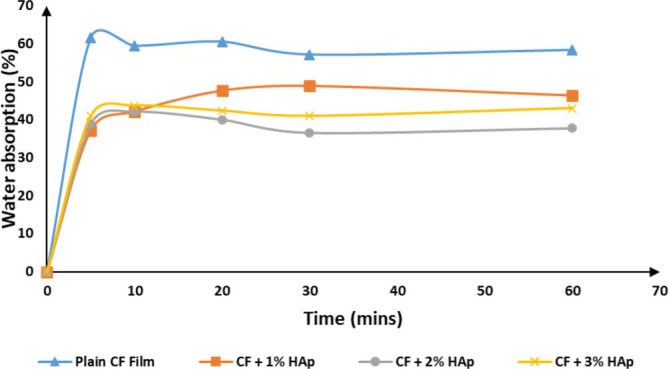
Water absorption kinetics of biopolymer composite films.

The water absorption kinetics of the biocomposite films are presented in Table 5, while the corresponding kinetic curves are depicted in Figure 13. These results illustrate the moisture stability of the biocomposite films by revealing the dynamics of water diffusion within their structure. The analysis showed that the neat cellulose film exhibited a higher initial water absorption compared to the FSHAp‐filled biopolymer composite films. The reduced rate of water uptake in the filler‐loaded films can be attributed to the presence of granular FSHAp particles, which possess inherent porosity and facilitate gradual water uptake and retention within their interstitial spaces. However, after 30 min, the biopolymer film containing 3 wt% FSHAp exhibited a marked increase in water absorption. This behavior is ascribed to insufficient matrix‐filler interactions in the film's microstructure. Water solubility is largely dictated by the polymer's molecular architecture and the strength of its intermolecular forces. The presence of cracks in the 3 wt% FSHAp‐filled film likely accelerated absorption kinetics, promoting pore formation and enabling sustained water accumulation within the polymer matrix. This outcome is in alignment with previous literature on the water absorption characteristic of biopolymer composite films [61]. Overall, the 2 wt% FSHAp‐filled biopolymer composite film demonstrated superior resistance to moisture ingress, likely due to improved matrix‐filler interfacial adhesion, which resulted in a denser, more compact structure evidenced by the absence of cracks and micro‐voids in the SEM micrograph (Figure 5c).

**TABLE 5:** Water absorption of biopolymer composite films.

Film sample	Dry weight (g)	After 5 min (g)	After 10 min (g)	After 20 min (g)	After 30 min (g)	After 1 h (g)
Plain CFs	0.13	0.26	0.30	0.34	0.32	0.31
CFs + 1% HAp	0.22	0.35	0.38	0.41	0.43	0.41
CFs + 2% HAp	0.34	0.54	0.57	0.55	0.52	0.53
CFs + 3% HAp	0.49	0.83	0.87	0.85	0.83	0.86

#### 4.7.4 Water Solubility (WS)

The water solubility analysis of the samples was conducted over a 48‐h period, with weight measurements taken at 24‐h intervals. The results, presented in Table 6, revealed that the neat cellulose film exhibited no solubility in water. This outcome aligns with previous findings, as cellulose, despite being hydrophilic and demonstrating a high capacity for water uptake [71], remains insoluble in water due to the presence of strong inter‐ and intramolecular hydrogen bonding within its polymeric network (Dai et al. [76]). After 24 h of immersion, the neat cellulose film reached saturation, beyond which no further water absorption was observed. In contrast, the FSHAp‐filled biocomposite films continued to absorb water beyond this period, primarily within the interstitial space of the porous FSHAp fillers. A comparison of the initial and final weights of the samples indicated that the biocomposite film containing 3 wt% FSHAp exhibited a notably higher weight loss. This phenomenon is attributed to inadequate matrix‐filler interactions at this concentration, which likely led to the separation of weakly bound filler particles. Optimal filler dispersion and strong interfacial bonding between the matrix and filler are critical for enhancing the water resistance of biopolymer composites. As reported by Che Aziz et al. [77], poor dispersion and aggregation of filler particles can significantly increase water uptake. This ultimately facilitates the removal of weakly incorporated fillers, contributing to the observed mass loss in the 3 wt% FSHAp‐filled film. The less smooth surface morphology observed for this sample further supports the presence of poor matrix‐filler interactions.

**TABLE 6:** Water solubility trend of biopolymer composite films.

Film sample	Initial dry weight (g)	Weight after 24 h (g)	Weight after 48 h (g)	Final weight after oven‐drying (g)
Plain CFs	0.15	0.35	0.35	0.15
CFs + 1% HAp	0.27	0.30	0.38	0.19
CFs + 2% HAp	0.32	0.52	0.55	0.22
CFs + 3% HAp	0.46	0.84	0.91	0.38

### 4.8 Coefficient of Friction

Table 7 presents the static and dynamic coefficient of friction of the biopolymer composite films. The coefficient of friction is a non‐dimensional quantity that characterizes the ratio between the frictional force and the normal force acting between two contacting surfaces. It provides a quantitative measure of the resistance encountered when one surface moves relative to another. This property is particularly critical for films intended for packaging applications, as it directly influences processing speed, equipment wear, and the overall efficiency of packaging operations. The coefficient of friction is typically reported in two forms: static and dynamic. The static coefficient of friction refers to the force required to initiate motion between two surfaces, whereas the dynamic (or kinetic) coefficient of friction refers to the force needed to sustain motion once it has begun. According to the literature, surface roughness impacts the coefficient of friction; rougher surfaces generally exhibit higher values (Bachchhav and Bagchi [78]; Dorn et al. [79]).

**TABLE 7:** The static and dynamic coefficient of friction of biopolymer composite films.

Film sample	Test speed (mm/min)	Static (*μ*_*s*_)	Dynamic (*μ*_*k*_)
Plain CFs	10	0.2339	0.2714
CFs + 1% HAp	10	0.3428	0.3724
CFs + 2% HAp	10	0.4582	0.4935
CFs + 3% HAp	10	0.6128	0.6533

In applications such as automotive tires, a high coefficient of friction typically within the range from 1.0 to 1.4 is essential for optimal performance and safety [80]. Conversely, in packaging applications, a lower coefficient of friction generally below 1.0 is preferred to facilitate smooth material handling and enhance processing efficiency [81]. In contrast, biopolymer hydrogels designed for biomedical applications may exhibit coefficients of friction as low as 0.1, which is beneficial for minimizing resistance in soft tissue environments [82]. In the present study, regenerated cellulose films (plain CFs) exhibited a static coefficient of friction of 0.23, indicating that a relatively low force is required to initiate movement across a surface. Based on this value, the film can be classified within the moderate slip category. Additionally, the dynamic coefficient of friction was recorded at 0.27, suggesting that the film offers minimal resistance once sliding has commenced. Dynamic coefficients of friction in the range of 0.3–0.4 are recorded for 1 and 2 wt% FSHAp filler concentrations. These are typically considered beneficial for reducing wear on machine components during processing. Notably, the biocomposite film containing 3 wt% FSHAp filler exhibited a dynamic coefficient of friction of 0.6, characterizing it as a non‐slip surface. Such a property may be advantageous in applications that require high resistance to sliding, and requiring enhanced grip. The increased surface roughness observed in the biopolymer composite film sample attributed to the presence of poorly incorporated FSHAp fillers at 3 wt% concentration contributed to its elevated coefficient of friction, as evidenced by the SEM micrograph (Figure 5d). Both the particle diameter and dispersion of fillers significantly influence the frictional behavior of composite films. Yücetürk et al. [83] reported that in bio‐based polyethylene films, surface micro‐roughness arising from the matrix‐filler interaction played a critical role in determining the frictional characteristics. Composites exhibiting microscopic attachment points on their surfaces demonstrated higher coefficients of friction, highlighting the importance of uniform filler dispersion and strong interfacial bonding in controlling surface friction.

## 5 Conclusion

The successful utilization of agricultural waste materials specifically banana fibers and fish scales as alternative sources to high‐grade cellulose and hydroxyapatite for the fabrication of cellulose‐based biopolymers with noteworthy thermal and mechanical properties has been demonstrated. The biopolymers were effectively dissolved in an ionic liquid to produce regenerated films, achieving a tensile strength of up to 23 MPa. Detailed analyses further confirmed effective interactions between the polymeric constituents of the films, particularly the electrostatic interaction between the positively charged calcium ions from hydroxyapatite and the negatively charged hydroxyl groups present in cellulose. Thermogravimetric analysis (TGA) revealed enhanced thermal stability, as evidenced by increased char formation and improved thermal resistance, attributable to the incorporation of FSHAp fillers. At 3 wt% FSHAp filler concentration, a 74.89% increase in char formation was observed compared to the regenerated cellulose film, indicating enhanced thermal stability attributable to the presence of the filler. The differential scanning calorimetry (DSC) results reveal that incorporating FSHAp fillers into the cellulose matrix alters the thermal stability by interrupting the chain packaging a structure. This study presents the development of a novel biopolymer composite derived from fish scale hydroxyapatite and cellulose. The use of biodegradable constituents, coupled with the inherent thermal stability and mechanical strength of FSHAp fillers and cellulose, makes this biopolymer composite a promising candidate for sustainable packaging applications. Future investigations should focus on assessing the durability and performance of the biocomposite films under mechanical stress and temperature conditions that simulate real‐world scenarios to better predict their applicability. Additionally, given that the water solubility of biopolymer films is largely governed by polymer structure and intermolecular interactions, the incorporation of environmentally friendly cross‐linking agents is recommended particularly at higher filler concentrations to enhance matrix‐filler interactions and overall structural integrity.

## Conflicts of Interest

The authors declare no conflicts of interest.

## Data Availability

The data that support the findings of this study are available from the corresponding author upon reasonable request.
